# Systematic Review and Meta-Analysis on Hysterectomy by Vaginal Natural Orifice Transluminal Endoscopic Surgery (vNOTES) Compared to Laparoscopic Hysterectomy for Benign Indications

**DOI:** 10.3390/jcm9123959

**Published:** 2020-12-07

**Authors:** Susanne Housmans, Nargis Noori, Supuni Kapurubandara, Jan J. A. Bosteels, Laura Cattani, Ibrahim Alkatout, Jan Deprest, Jan Baekelandt

**Affiliations:** 1 Department of Development and Regeneration, Faculty of Medicine, Group Biomedical Sciences, KU Leuven, 3000 Leuven, Belgium; jan.bosteels@imelda.be (J.J.A.B.); laura.cattani@uzleuven.be (L.C.); jan.deprest@uzleuven.be (J.D.); 2 Department of Obstetrics and Gynaecology, University Hospitals Leuven, 3000 Leuven, Belgium; 3 Department of Obstetrics and Gynaecology, Westmead Hospital, Westmead 2145, Australia; nargisn24@gmail.com (N.N.); supuni_c_k@yahoo.co.uk (S.K.); 4 Sydney West Advanced Pelvic Surgery (SWAPS), Bella Vista 2153, Australia; 5 Department of Obstetrics and Gynaecology, University of Sydney (USYD), Sydney 2052, Australia; 6 Department of Obstetrics and Gynaecology, Imelda Hospital, 2820 Bonheiden, Belgium; 7 Kiel School of Gynaecological Endoscopy, Department of Gynaecologiy and Obstetrics, University Hospitals Schleswig-Holstein, Campus Kiel, 24105 Kiel, Germany; Ibrahim.alkatout@uksh.de

**Keywords:** hysterectomy, NOTES, laparoscopy, minimally invasive, systematic review, meta-analysis

## Abstract

(1) Objective: We aimed to report an update of the systematic review and meta-analysis by Baekelandt et al. (2016). (2) Method: We followed PRISMA guidelines to perform this systematic review. We searched MEDLINE, EMBASE, CENTRAL and additional sources and aimed to retrieve randomised controlled trials (RCTs), controlled clinical trials (CCTs) and prospective/retrospective cohort studies in human subjects that allowed direct comparison of vNOTES to laparoscopy. (3) Results: Our search yielded one RCT and five retrospective cohort trials. Pooled analysis of two subgroups showed that, compared to conventional laparoscopy, vNOTES is equally effective to successfully remove the uterus in individuals meeting the inclusion criteria. vNOTES had significantly lower values for operation time, length of stay and estimated blood loss. There was no significant difference in intra- and postoperative complications, readmission, pain scores at 24 h postoperative and change in hemoglobin (Hb) on day 1 postoperative.

## 1. Introduction

### Background and Rationale

In natural orifice transluminal endoscopic surgery (NOTES), the natural orifices of the human body are used to access the abdominal cavity to perform surgery. Since the safety of colpotomy is not debated, transvaginal NOTES was the first to be adopted clinically, not only for hysterectomy but also for adnexal and even gastro-intestinal surgery such as cholecystectomy and appendectomy [1]. The proposed benefits of NOTES include reduced postoperative pain, faster postoperative recovery and improved cosmesis compared to standard laparoscopic approaches using the abdominal wall as access [2]. Hysterectomy via vaginal natural orifice transluminal endoscopic surgery (vNOTES) was first described by Su et al. in 2012 [3]. The route through which hysterectomy for benign disease is performed is determined by many factors including the size of the uterus, accessibility to the uterus, extra uterine disease, patient preference and surgeon preference and training. Current evidence supports vaginal hysterectomy as superior to laparoscopic and abdominal routes due to the shorter operating time and shorter recovery time [4]; however, its clinical application can be restricted by poor visualisation and limited manipulation [5]. These restrictions might be overcome with vNOTES as it combines the advantages of vaginal and endoscopic surgery [6]. An earlier systematic review and meta-analysis by Baekelandt et al. [2] assessed the effectiveness and safety of vNOTES hysterectomy for non-prolapsed uteri and benign gynecological disease compared to the conventional laparoscopic technique. As many publications including a randomised control trial have been published since, we aimed to update this systematic review.

The objectives of this systematic review are to assess the effectiveness and safety of vNOTES hysterectomy for non-prolapsed uteri and benign gynecological disease compared to conventional laparoscopic techniques.

1. Is vNOTES equally effective as the laparoscopic approach for successful removal of the uterus without the need for conversion?
2. Is the operation time for removal of the uterus by vNOTES faster compared to laparoscopy?
3. Is the complication rate of vNOTES hysterectomy different compared to laparoscopy?
4. What is the difference in hospital stay in women treated by vNOTES compared to laparoscopy?
5. What is the readmission rate in women after hysterectomy by vNOTES versus by conventional laparoscopy?
6. What is the difference in postoperative pain between women treated by vNOTES hysterectomy and conventional laparoscopic hysterectomy?
7. Are there differences in women’s health, concerning dyspareunia, sexual wellbeing or health-related quality of life after hysterectomy by vNOTES compared to laparoscopy?
8. Are there differences in the financial costs of both techniques?

## 2. Methods

We conducted this systematic review according to the Cochrane Handbook for Systematic Reviews [7] and reported following PRISMA guidelines [8]. The protocol of this review was registered in PROSPERO under registration number CRD42020198104.

### 2.1. Eligibility Criteria

We aimed to retrieve randomised controlled trials (RCTs), controlled clinical trials (CCTs) and prospective/retrospective cohort studies in human subjects that allow direct comparison of vNOTES to laparoscopy. All studies that did not allow direct comparison (e.g., case series, case reports, editorials, letters to the editor) were excluded. There was no restriction in timeframe or language, provided that articles could be translated using Google Translate if necessary.

### 2.2. Population

We included studies in the adult female population, undergoing removal of the uterus for benign gynecological disease. Studies on interventions for genital prolapse or gynecological malignancy were excluded.

### 2.3. Intervention

vNOTES hysterectomy was the experimental intervention.

### 2.4. Comparison

Hysterectomy by conventional laparoscopy using the umbilicus was the comparator. This included laparoscopy assisted vaginal hysterectomy (LAVH), total laparoscopic hysterectomy (TLH) using single port (SILS) or multiple port (MP) access. We excluded abdominal and vaginal hysterectomy as comparator.

### 2.5. Outcome

Primary outcome was the proportion of women successfully treated with the intended approach to perform hysterectomy without conversion to any other technique

Secondary outcomes:

1. Duration of surgery (in minutes).
2. Intra- or postoperative complications using the Clavien–Dindo classification [9,10] and postoperative infection defined by lower abdominal pain with fever (>38°) and suggestive clinical signs or laboratory findings.
3. Length of hospital stay in days.
4. Readmission after discharge.
5. Postoperative pain measured by visual analogue scale (VAS).
6. Women’s health reported as incidence and severity of dyspareunia, sexual wellbeing and quality of life (QOL) measured by validated tools.
7. Comparative financial cost.

### 2.6. Literature Search

We developed a search strategy by combining medical subject headings (MeSH, Emtree) and free text words. The complete search strategy for all databases is presented in Appendix A.

The final literature search was done until 8 October 2020. We searched MEDLINE (PubMed interface), EMBASE, the Cochrane Central Register of Controlled Trials (CENTRAL). Additional sources we searched were ClinicalTrials.gov, the WHO ICTRP search portal, Web of Science, INAHTA, LILACS and Open Grey. The search of the Centre for Reviews and Dissemination (CRD) used in the first review was preplaced by that of INAHTA, as the two other databases in CRD (DARE and NHS EED) have not been updated since 2015 and are hence not relevant for the search strategy of this systematic review.

### 2.7. Study Selection

Two independent reviewers (SH and NN) screened the titles and abstracts and obtained full text reports of all titles that met the inclusion criteria. After screening the full text records, any disagreement was resolved by a third reviewer (JJAB).

### 2.8. Data Collection

Two reviewers (SH and NN) extracted data from the eligible studies using standardised data extraction forms. Data were extracted for: study design, study population, in- and exclusion criteria, interventions, comparators and outcomes. We calculated mean values and standard deviation (SD) if these were expressed as median and range for continuous data. The study authors were contacted to resolve uncertainties.

### 2.9. Risk of Bias Assessment

We aimed to assess the methodological quality of the selected studies by applying the RoB2 tool to assess the risk of bias in randomised trials [11] and the ROBINS-I tool for non-randomised trials [12]. The risk of bias assessment was performed by two reviewers independently (SH and NN) and disagreement was resolved by discussion and when needed by consulting a third review author (JJAB). We aimed to assess bias across studies for each outcome measure and pool data based on study design.

### 2.10. Summary Measures

Continuous data were analysed as mean differences (MD) with a 95% confidence interval (CI). We analysed ordinal outcomes as continuous outcomes. Dichotomous data were reported as an odds ratio (OR) with a 95% CI.

### 2.11. Synthesis of Results

For the meta-analysis, we combined each outcome and calculated the summary effect size using Review Manager 5.4 software (http://training.cochrane.org). We used the Mantel–Haenszel method (M-H) for the fixed effect model for dichotomous data and Inverse Variance (IV) for the fixed effect model for continuous data. Subgroup analysis was done to compare randomised and observational studies. When possible, heterogeneity was tested by the I^2^ test. Overall effect was reported as Z-score where *p* value < 0.05 was considered significant.

## 3. Results

**Figure 1:**
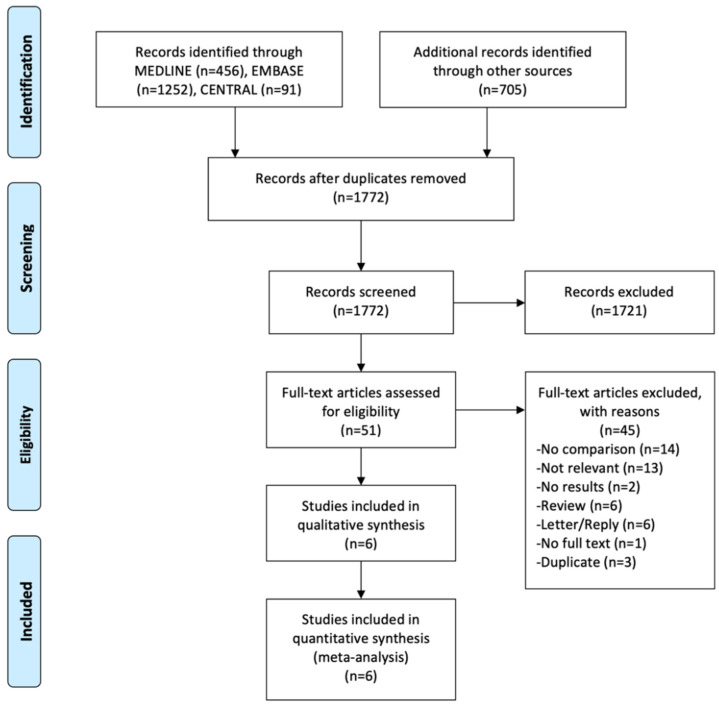
PRISMA flow chart.

In total, we retrieved 2504 records. MEDLINE, EMBASE and CENTRAL yielded 1799 records. The additional search described above added another 705 records. After removing duplicates (*n* = 732) in Endnote X9 (Clarivate Analytics, Philadelphia, PA, USA), 1772 records were uploaded in Rayyan (http://rayyan.qcri.org) and screened by title and abstract. Full text screening for eligibility was done for the remaining 51 records and six records were included in the systematic review (Figure 1).

### 3.1. Description of the Studies

We refer to Table 1 and Table S1 for more detailed characteristics of the included studies.

We retrieved one RCT and five observational studies that allowed for direct comparison between vNOTES hysterectomy and conventional laparoscopic hysterectomy.

The HALON trial by Baekelandt et al. [13] was the only published RCT that we could retrieve at the moment of this review, although we found registrations of two planned or ongoing RCTs during our search [14,15]. The HALON trial was conducted at Imelda Hospital in Belgium, from December 2015 to June 2017. The study group consisted of 70 women aged 34–68 years old who were scheduled for hysterectomy for benign disease. Study participants were randomly assigned in a 1:1 fashion to vNOTES with superficial abdominal skin incisions to allow blinding (experimental group) or TLH (control group). All surgical procedures were done by the same surgeon. Primary outcome was hysterectomy by the allocated technique. Secondary outcomes were the number of patients leaving the hospital within 12 h (day care setting), length of hospital stay, occurrence of complications, total use of analgesics, postoperative visual analogue scale (VAS) pain scores, direct health care costs, dyspareunia and quality of life (QoL).

The study by Wang et al. [16] is a retrospective cohort study conducted in 2015 at Chang Gung Memorial Hospital in Linkou, Taiwan. The study group consisted of 147 women aged 38–69 years with different indications scheduled to undergo hysterectomy by vNOTES between April 2011 and October 2013. The comparison group consisted of 365 women receiving LAVH. All surgical procedures were done by the same surgeon. The authors used a propensity score matched analysis: the sample of 147 vNOTES cases was compared with a similar number of LAVH treated women group using a “nearest neighbour” approach. The following outcomes were studied: the operative time, the estimated blood loss, complications, the length of postoperative hospital stay and the hospital charges.

The study by Yun Seok Yang et al. [17] is a retrospective cohort study conducted in 2014 at Eulji University Hospital in Doonsandong Daejeon, South Korea. The study group consisted of 16 women undergoing hysterectomy by vNOTES between July 2012 and June 2013. The comparison group consisted of 32 women undergoing hysterectomy by single port LAVH (SP-LAVH) during the same study period and who were matched by age, body mass index (BMI), parity, number of previous abdominal surgeries and weight of uterus. All surgical procedures were done by the same surgeon. The following outcomes were measured: operative time, estimated blood loss, complications, length of postoperative hospital stay, decrease in hemoglobin on postoperative day one and the total amount of analgesics used.

The study by Kim et al. [18] is a retrospective cohort study conducted in 2017 at Eulji University Hospital in Doonsandong Daejeon, South Korea. The study group consisted of 40 women undergoing vNOTES hysterectomy (in this article referred to as NAVH—natural orifice transluminal endoscopic surgery-assisted vaginal hysterectomy) between July 2012 and September 2015. These subjects were matched in terms of baseline characteristics (age, height, weight, BMI), with 120 patients undergoing conventional 3-port LAVH. The surgical procedures were done by the same team. The following outcomes were measured: operation time, complications, uterine weight, hemoglobin change between preoperative and postoperative day 1.

The retrospective cross-sectional study was conducted by Kaya et al. [19] in 2020 at the University of Health Sciences, Bakirkoy Dr. Sadi Konuk Training and Research Hospital in Istanbul, Turkey. During the time period reviewed, between January 2016 and 2019, the study group consisted of 30 patients that underwent vNOTES hysterectomy for various benign reasons. The control group consisted of 69 patients that underwent TLH during the same period. In the control group, 30 records were matched with the study group with a multiple logistic propensity score-matching analysis. All the surgical procedures were performed by the same surgeon. The following outcomes were measured: operating time, length of stay, VAS scores at the 6th and 24th hours, decrease in Hb/Hct and complications.

The study by Chih-Yi Yang et al. [20] is a retrospective study conducted in 2020 at the China Medical University Hospital in Taiwan. The study group consisted of 20 patients that underwent vNOTES hysterectomy for benign, non-prolapse indications between January 2015 and December 2017. The control group consisted of 66 patients that underwent TLH in the same period. All the surgical procedures were performed by the same surgeon. The following outcomes were measured: operation time, blood loss during surgery, uterine weight, decrease in Hb level on postoperative day 1, postoperative pain scale (VAS), postoperative complications, length of stay and re-admission rate.

### 3.2. Risk of Bias within Studies

The RoB2 tool [11] was used to assess the risk of bias in the RCT (HALON trial). In this trial, the risk of bias was considered low. The five other included studies were observational studies, based on retrospective chart analysis, assessed for bias with the ROBINS-I tool [12]. The risk of bias was moderate, which can be attributed to the retrospective design of the studies, leading to selection bias and bias on measurement of outcomes and publication bias. A summary of the risk of bias assessment is presented in Table 2.

### 3.3. Results of Individual Studies

Details of the individual results can be found in Table 1 and Table S1.

1. Is vNOTES equally effective as the laparoscopic approach for successfully removing the uterus without the need for conversion? The HALON trial [13] is an RCT designed to answer this question as the primary outcome. No conversions were reported. Neither did the studies by Y. S. Yang et al. [17] and Kaya et al. [19]. Kim et al. [18] reported one conversion in the experimental group, but the reason for conversion is not mentioned. The studies by Wang et al. [16] and C-Y. Yang [20] do not explicitly mention conversions in their cohorts.
2. Duration of surgery. Except for the study by Kim et al. [18], all included studies reported a shorter operation time for vNOTES compared to LAVH, TLH or SP-LAVH. This result was significant in each study except for the study by C-Y. Yang [20].
3. Intra- or postoperative complications using the Clavien–Dindo classification [9,10] and postoperative infection defined by lower abdominal pain with fever >38° and suggestive clinical signs or laboratory findings are summarized in Table 3. Clavien–Dindo score is reported in parentheses.
4. Length of stay. Four studies (Wang et al. [16], Y. S. Yang et al. [17], Baekelandt et al. [13] and Kaya et al. [19]) showed a significantly shorter length of hospital stay after vNOTES compared to their control. The other two studies did not report a significant difference.
5. Readmission after discharge. Four studies reported on readmission after discharge. Wang et al. [16] reported one readmission in the control group due to vault hematoma. S. Y. Yang et al. [17] reported no readmissions. Baekelandt et al. [13] reported one readmission in the vNOTES group (suspicion of deep venous thrombosis (DVT) demanding CT angiography) and six in the control group (two for pain, one for cuff infection, one for vault hematoma, one for repair of a vesicovaginal fistula and one for pulmonary embolism with ICU admission). C-Y. Yang et al. [20] reported three readmissions in the control group due to pelvic inflammatory disease (PID). None of these findings was significant in the individual reports.
6. Postoperative pain measured by visual analogue scale (VAS). Four studies reported on postoperative pain scores by VAS. Y.S. Yang et al. [17] reported pain scores at 12 and 24 h postoperative. VAS scores at 12 h were 2 (range 0–6) for vNOTES and 2 (0–6) for LAVH. VAS scores at 24 h were 0 (0–4) for vNOTES and 0.5 (0–8) for LAVH. None of these differences were significant. Baekelandt et al. [13] reported pain scores twice a day in the first week after surgery. Average VAS pain score was consistently and significantly lower in the vNOTES group compared to TLH. We requested and received the VAS scores on postoperative day 1 to use for this meta-analysis. Kaya et al. [19] reported VAS scores at 6 and 24 h postoperative. VAS pain score at 6 h was 6 (range 4–7) for vNOTES and 6 (3–7) for TLH. Scores at 24 h were 2 (2–4) for vNOTES and 2 (0–5) for TLH. Differences were not statistically significant. C-Y. Yang et al. [20] reported significantly lower postoperative pain scores comparing vNOTES to TLH.
7. Incidence and severity of dyspareunia, sexual wellbeing and quality of life (QOL) measured by validated tools. Only Baekelandt et al. [13] report on this outcome. They report no differences between both arms of the RCT for occurrence and severity of pain on sexual intercourse at 3 and 6 months and health related quality of life at 3 and 6 months.
8. Comparative financial cost. Two studies mention financial cost. The study by Wang et al. [16] reported significantly higher hospital charges for vNOTES compared to LAVH: 22,573.3 +/− 5528.8 vs. 17,744.6 +/− 8939.2 New Taiwan Dollar (NTD). They mention that this was driven by the higher cost of disposable devices (wound retractor and vessel sealing device) in spite of a shorter hospital stay for vNOTES. Baekelandt et al. [13] reported no difference in direct health-related cost by measuring the difference in hospital bill up to 6 weeks postoperative. The direct hospital charge for disposable devices is not reflected entirely in the hospital bill described in the latter report, as the Belgian national health insurance automatically covers the cost of disposable devices up to approximately 550 EUR.

### 3.4. Synthesis of Results

Our search for studies allowing a direct comparison between vNOTES hysterectomy and conventional laparoscopic hysterectomy yielded six studies: one RCT and five observational studies. In each study, the interventions in both comparison arms were performed by one surgeon or one team, either during or beyond their learning curve for vNOTES. Although the control groups varied in type of surgery (TLH, LAVH or SP-LAVH) and the technique for the vNOTES approach is not standardised across different studies, we considered it useful to pool the data into a meta-analysis comparing the results of the RCT to those of the observational studies. The pooled results for the different outcomes are described here.

**Figure 2:**
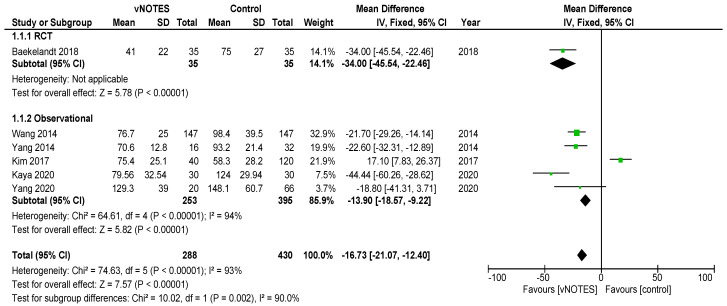
Forest plot of comparison vNOTES versus control, outcome: operation time.

**Figure 3:**
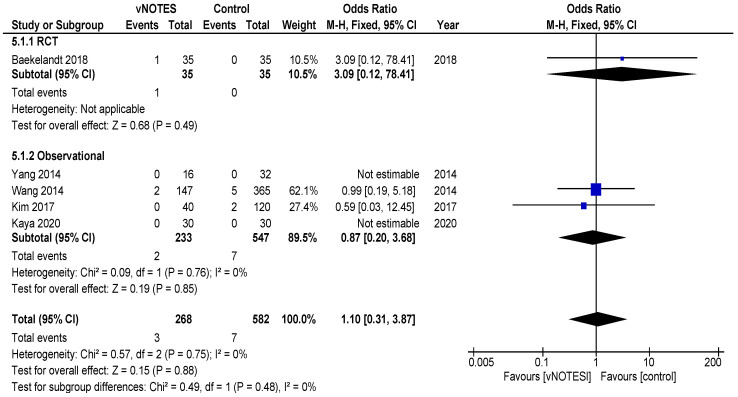
Forest plot of comparison vNOTES versus control, outcome: intraoperative complications.

**Figure 4:**
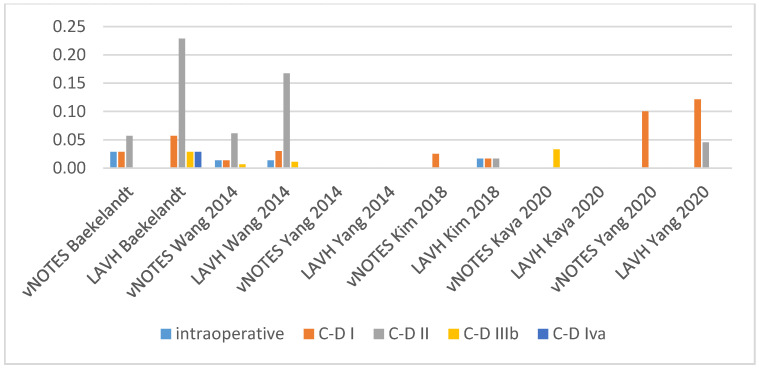
Intra- and postoperative complications (C-D: Clavien–Dindo score).

**Figure 5:**
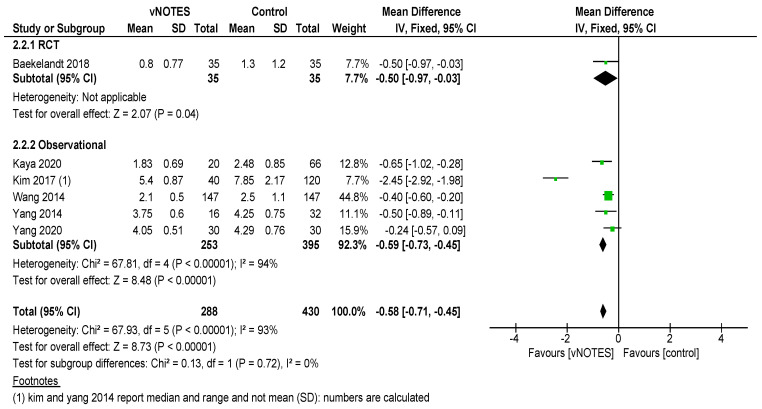
Forest plot of comparison vNOTES versus control, outcome: length of hospital stay.

**Figure 6:**
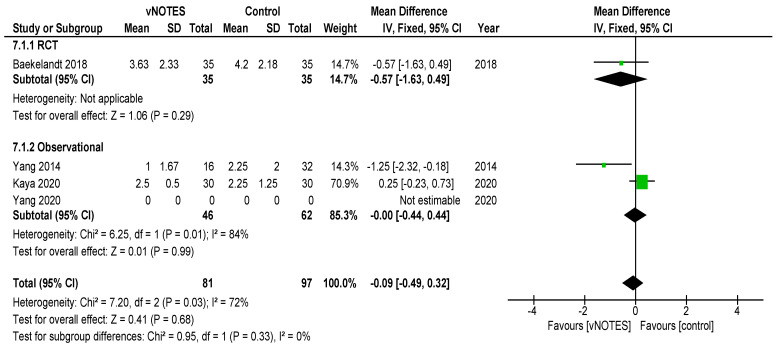
Forest plot of comparison vNOTES versus control, outcome: VAS pain scores Day 1.

1. Is vNOTES equally effective as the laparoscopic approach for successfully removing the uterus without the need for conversion? Zero or very few conversions were reported in the studies examined for this review. Keeping in mind possible selection bias in the observational studies and case selection applied in all reports reviewed, we consider vNOTES equally effective.
2. Is the operation time (OT) of hysterectomy by vNOTES shorter compared to laparoscopy? The pooled data showed a mean difference in operation time (OT) of 16.73 min, in favour of vNOTES (MD −16.73 (95% CI −21.04 to −12.40), Z = 7.57 (*p* < 0.05)) (Figure 2). We performed a sensitivity analysis on the outlier, the study by Kim et al. [18], which is responsible for the high heterogeneity in this subgroup. They reported a significantly shorter OT for the control group. We believe this to be attributed to the technique of LAVH described in the paper, where the dissection of the ovarian ligaments, round ligaments and broad ligaments was performed by a 45 mm EndoGia^®^ (Covidien, Ireland).
3. Is the complication rate of vNOTES hysterectomy different compared to laparoscopy? The types of complications reported were comparable across studies and comparison arms. Intraoperative complications were of bladder or bowel injury or bleeding in both vNOTES and laparoscopic hysterectomy. The differences were not significant (OR 1.10 (95% CI 0.31 to 3.87)) (Figure 3). Postoperative infection (reported as fever or PID) was less frequent in vNOTES than in controls (OR 0.41 (95% CI 0.17 to 0.99), Z = 1.98 (*p* = 0.05) (Figure S1). Figure 4 shows the fraction of intra- and postoperative infections according to the Clavien–Dindo classification [9,10]. Clavien–Dindo grade I contains cases of fever (without mentioning treatment with antibiotics), pain and hematoma. Grade II contains cases of wound infections, PID and blood transfusion. Grade IIIb contains one case of vesicovaginal fistula repair and cases of reintervention for bleeding. The case in Grade IVa is a case of pulmonary embolism with ICU admission (summary in Table 3). The pooled data for postoperative complication show an OR of 0.38 (95% CI 0.23 to 0.62) in favour of vNOTES. This result was not significant (Figure S2). We additionally pooled data on estimated blood loss (EBL) and decrease in Hb on postoperative day 1 (Figures S3 and S4). EBL was significantly lower in vNOTES (MD −98.87 mL (95% CI −126.67 to −71.07), Z = 6.97 (*p* < 0.05).
4. What is the difference in hospital stay in women treated by vNOTES compared to laparoscopy? Although there was substantial variation in mean hospital stay between studies, hospital stay was shorter for vNOTES in each study. The pooled data showed a mean difference (MD) of 0.58 days (95% CI −0.71 to −0.45) in favour of vNOTES. Z = 8.73 (*p* < 0.05). We performed a sensitivity analysis for the outlier, the study by Kim et al. [18], which is responsible for the high heterogeneity in this subgroup. They report a range of 4–17 days in length of stay for their control group, leading to a higher MD, which was calculated from the reported median (Figure 5).
5. What is the readmission rate in women after hysterectomy by vNOTES versus by conventional laparoscopy? Pooled analysis of the reported readmissions showed a lower rate of readmissions after vNOTES (OR 0.18 (95% CI 0.03 to 1.08)). This difference was not significant (Figure S5).
6. What is the difference in postoperative pain between women treated by vNOTES hysterectomy and conventional laparoscopic hysterectomy? The randomised trial by Baekelandt et al. [13] reports lower pain scores (VAS 0–10) at 24 h postoperative (not significant), which is comparable to the pooled results extracted for three studies (MD −0.09 (95% CI −0.49 to 0.32)). The data from Chin-Yi Yang et al. [20] could not be used for the pooled analysis for this outcome as we were unable to retrieve information on the timepoint of this score after surgery. (Figure 6).
7. Are there differences in women’s health after hysterectomy by vNOTES compared to laparoscopy concerning dyspareunia, sexual wellbeing or health-related quality of life? No pooled data were available since only one study reported on this outcome [13].
8. Are there differences in the financial costs of both techniques? The results of the two studies [13,16] reporting this outcome measure are too heterogenous to allow pooling of the data.

## 4. Discussion

### 4.1. Summary of Evidence

The pooled results of the reports that we selected show that vNOTES is equally effective as conventional laparoscopy in successfully removing the uterus in individuals meeting the inclusion criteria. vNOTES had significantly lower values for operation time, length of stay and estimated blood loss. There was no significant difference in intra- and postoperative complications, readmission, pain scores at 24 h postoperative and change in hemoglobin (Hb) on day 1 postoperative. We were unable to perform meta-analysis on the outcomes on women’s health and comparative cost.

### 4.2. Limitations

Since our search yielded only six studies, of which only one was an RCT, the strength of evidence is low. The results of the RCT are in line with those of the observational studies. The quality of the observational trials is limited due to the non-random allocation of patients. To reduce the risk for selection bias in the observational studies, two studies [16,19] used matched controls based on baseline characteristics, whereas two other studies [17,18] applied propensity score matching. Although Chin-Yi Yang et al. [20] did not report any correction for bias, the baseline characteristics in both groups were comparable. All interventions were done by experienced endoscopic surgeons, but for vNOTES, some authors report not being beyond their learning curve. All studies involved a single surgeon or single surgical team, and the included studies are concentrated in predominantly Asian centres. This may limit the generalisability of the results.

## 5. Author’s Conclusions

We aimed to perform a systematic review comparing vNOTES hysterectomy to conventional laparoscopic hysterectomy. Six studies were included in the meta-analysis. The available randomised and observational data show that vNOTES hysterectomy is an effective and safe novel technique for women eligible for endoscopic surgery. Further prospective multicentre randomised trials are needed which are designed to include outcomes on financial cost and women’s health. Our search yielded two ongoing trials [14,15]. Although our scope was to select studies on hysterectomy for benign disease, many IDEAL stage 1 studies indicate the use of vNOTES for other gynaecological surgery. These studies report on the use of vNOTES for benign indications (adnexal surgery, myomectomy, prolapse surgery, and so on) and for oncologic indications (borderline ovarian cancer and endometrial cancer). No randomised controlled trials for these indications have been published to date.

## Figures and Tables

**Table 1:** Characteristics of the included studies.

Item	Wang 2014	Y S Yang 2014	Kim 2018	Baekelandt 2019	Kaya 2020	C-Y Yang 2020
study design	Retrospective chart analysis (Canadian Task Force Classification II-1)	Retrospective chart analysis (Canadian Task Force Classification II-1)	Retrospective chart analysis (Canadian Task Force Classification II-1)	RCT, non-inferiority trial, single blind	Cross-sectional study (retrospective)	Retrospective chart analysis (Canadian Task Force Classification II-1)
study setting	Single centre tertiary referral hospital	Single centre university affiliated hospital	Single centre university affiliated hospital	Single centre, teaching hospital	Single centre university affiliated hospital	Single centre university affiliated hospital
population	Women undergoing hysterectomy for benign uterine diseases in a non-prolapsed uterus aged 38–69 years Intervention: tVNOTEH (*n* = 147) Control: LAVH (*n* = 365)	Women undergoing hysterectomy for benign uterine diseases Intervention: NAVH (*n* = 16) Control: SP-LAVH (*n* = 32)	Women undergoing surgery for benign uterine disease, NAVH (*n* = 40), LAVH (*n* = 120)	Women 18–70 years old, undergoing hysterectomy for benign disease. vNOTES (*n* = 35), TLH (*n* = 35)	Women undergoing hysterectomy (TLH or vNOTES) for various gynaecological indications. vNOTES (*n* = 30), TLH (*n* = 69)	All women (*n* = 183, aged 38–56 years) undergoing TLH or vNOTES during the study period vNOTES (*n* = 31) and TLH (*n* = 152)
inclusion criteria	The indications for surgery in these patients included uterine myomas, adenomyosis, severe cervical dysplasia and menometrorrhagia.	Patients with benign uterine diseases documented by results from ultrasound examinations and who fulfilled the inclusion criteria, which included no history of pelvic inflammatory disease or medical illness.	Benign uterine disease, such as uterine myoma, adenomyosis, endometriosis	Age 18–70, benign disease such as symptomatic uterine fibroids, adenomyosis, high-grade cervical dysplasia, treatment-refractory dysfunctional uterine bleeding, atypical endometrial hyperplasia, BRCA-positive women 45 years or older	Various gynaecological indications, such as adnexal masses, uterine fibroids and treatment resistant heavy menstrual bleeding	Patients undergoing hysterectomy who did not have any of the below mentioned exclusion criteria
exclusion criteria	For tVNOTEH: history of abdominal–pelvic surgery with adhesion formation suspected, uterine prolapsed (international continence society classification Stage III or IV), suspected severe endometriosis and complete obliteration of the posterior Douglas pouch noted at pelvic examination. A history of cesarean section and nulliparity were not considered as contraindications for tVNOTEH.	History of pelvic inflammatory disease or medical illness, history of severe adhesions, suspected severe endometriosis, suspicion of gynaecologic malignancy or a fixed uterus and strong pelvic adhesions noted at pelvic examination were excluded.	Diagnosis of malignancy, findings of severe pelvic adhesions or a fixed uterus	History of rectal surgery, suspected rectovaginal endometriosis, suspected malignancy, pelvic inflammatory disease (PID), active lower genital tract infection, virginity, pregnancy	Presence of sacro-uterine nodularities, tubo-ovarian abcesses, endometriosis, pregnancy, history of colorectal surgery, uterine size above the umbilicus, uterine immobility in pelvic examination, suspicion of uterine sarcoma and any other pelvic organ malignancy	Prolapsed uterus, history of previous abdominal surgery, possibility of gynaecological malignancy, severe pelvic adhesions and a fixed uterus as determined by vaginal examination
intervention	tVNOTEH	NAVH	NAVH	vNOTES hysterctomy (VANH) plus 4 superficial non-therapeutic skin incisions identical to those in the control group	vNOTES hysterectomy	vNOTES hysterectomy
comparison	LAVH	SP-LAVH	LAVH	TLH	TLH	TLH
outcomes	Operating time, estimated blood loss, postoperative Hb, complication, postoperative stay, requirement of blood transfusion and hospital charges	Operating time estimated blood loss, decrease in hemoglobin on POD1, amount of analgesic drugs used, intra- and postoperative complications and length of postoperative hospital stay.	Operating time, intraoperative/postoperative complications, uterine weight, hemoglobin change between preoperative and postoperative day 1	Primary: removal of the uterus according to the allocated technique Secondary: operation time, proportion of hospital stay <12 h, length of hospital stay, complications, total amount of analgesics used, VAS pain scores in the first week, costs on the hospital bill until 6 weeks postoperative, occurrence and severity of dyspareunia before and 3 and 6 months after surgery, QOL at baseline and 3 and 6 months after surgery	Operating time, change in Hb and Hct, peri- and postoperative complications, length of hospital stay, conversion, VAS score at 6 and 24 h postoperative	Operating time, blood loss, decrease in hemoglobin level POD1, postoperative complication, length of stay, re-admission rate
*n* (vNOTES)	147	16	40	35	30	20
*n* (control)	147	32	120	35	30	66
operative time (min)	tVNOTEH 76.7 +/− 25.0 vs. LAVH 98.4 +/− 39.5	NAVH 70.6 +/− 12.8 SP-LAVH 93.2 +/− 21.4	NAVH 75.4 +/− 25.1 vs. LAVH 58.3 +/− 28.2	vNOTES 41 +/− 22 vs. TLH 75 +/− 27	vNOTES 79.56 +/− 32.54 vs. TLH 124 +/− 29.94	vNOTES 129.3 +/− 39.0 vs. TLH 148.1 +/− 60.7
estimated blood loss (EBL) (mL)	191.8 +/− 201.3 vs. 324.6 +/− 242.4	201.8 +/− 127 vs. 228.1 +/− 172	NI	NI	NI	53.5 +/− 74.9 vs. 43.8 +/− 83.7
decrease in Hb (g/L)	NI	1.05 +/− 1.06 vs. 1.42 +/− 1.07	0.975 +/− 0.826 vs. 1.339 +/− 1.057 (corrected after consulting author)	NI	1.1 +/− 0.9 vs. 0.84 +/− 0.93	1.14 +/− 0.6 vs. 1.09 +/− 0.75

NI: no information, tVNOTEH: transvaginal natural orifice transluminal endoscopic hysterectomy, LAVH: laparoscopy-assisted vaginal hysterectomy, NAVH: NOTES-assisted vaginal hysterectomy, SP-LAVH: single port laparoscopy assisted vaginal hysterectomy, TLH: total laparoscopic hysterectomy, LH: laparoscopic hysterectomy, AH: abdominal hysterectomy, POD1: postoperative day 1.

**Table 2:** Simplified summary of the risk of bias assessment.

	Baekelandt 2018	Wang 2014	Yang 2014	Kim 2018	Kaya 2020	Yang 2020
Bias due to confounding	low risk	moderate risk	moderate risk	moderate risk	moderate risk	moderate risk
Selection Bias	low risk	moderate risk	moderate risk	moderate risk	moderate risk	moderate risk
Bias in classification of interventions	low risk	low risk	low risk	low risk	low risk	low risk
Bias due to deviations from intended intervention	low risk	low risk	low risk	low risk	low risk	low risk
Bias due to missing data	low risk	low risk	low risk	low risk	low risk	low risk
Bias in measurement of outcomes	low risk	low risk	moderate risk	moderate risk	moderate risk	moderate risk
Bias in selection of reported result	low risk	low risk	low risk	low risk	low risk	low risk
Overall	low risk	moderate risk	moderate risk	moderate risk	moderate risk	moderate risk

**Table 3:** Summary of complications in the individual studies.

Study	Complications in vNOTES	Complications in Control
**Wang 2014**	*n* = 147 *intraoperative* 1 bleeding 1 bladder trauma *postoperative * 9 transfusions (II) 2 fever (I) 1 reintervention for bleeding (IIIb)	*n* = 365 *intraoperative* 3 bleeding 1 ureter trauma 1 bowel trauma *postoperative * 61 transfusions (II) 10 fever (I) 4 reinterventions for bleeding (IIIb) 1 vault hematoma (I)
**Yang 2014**	*n* = 16 *intraoperative* 0 *postoperative * 0	*n* = 32 *intraoperative* 0 *postoperative * 0
**Kim 2018**	*n* = 40 *intraoperative* 0 *postoperative* 1 fever (I)	*n* = 120 *intraoperative* 1 bladder and vagina trauma 1 bowel trauma *postoperative* 2 fever (I) 2 bleeding (II)
**Baekelandt 2018**	*n* = 35 *intraoperative* 1 bladder trauma *postoperative* 1 suspicion DVT (I) 1 infected hematoma (II) 1 transfusion (II)	*n* = 35 *intraoperative * 0 *postoperative* 2 pain (I) 2 vaginal cuff infection (II) 1 hematoma (I) 4 UTI (II) 1 transfusion (II) 1 ileitis (II) 1 repair vesicovaginal fistula (IIIb) 1 pulmonary embolism (IVa)
**Kaya 2020**	*n* = 30 *intraoperative* unclear * postoperative unclear *	*n* = 30 *intraoperative* unclear * postoperative unclear * 1 reintervention for bleeding (IIIb)
**Yang 2020**	*n* = 20 *intraoperative* NI ** postoperative 2 fever (I)	*n* = 66 *intraoperative* NI ** postoperative 8 fever (I) 3 PID (II)

* The report mentions bleeding and transfusion but does not state number of events nor in which arm. ** NI: no information.

## Supplementary Materials

The following are available online at https://www.mdpi.com/2077-0383/9/12/3959/s1, Table S1: Characteristics and outcomes of the included studies (supplementary to Table 1); Figure S1: Forest plot of comparison vNOTES versus control, outcome: infections; Figure S2: Forest plot of comparison vNOTES versus control, outcome: postoperative complications; Figure S3: Forest plot of comparison vNOTES versus control, outcome: Estimated blood loss (EBL); Figure S4: Forest plot of comparison vNOTES versus control, outcome: Change in hemoglobin (Hb) preoperative-postoperative day 1; Figure S5: Forest plot of comparison vNOTES versus control, outcome: Readmission after discharge.

## Appendix A. Complete Search Strategy

Pubmed

(“Hysterectomy” [Mesh] OR hysterectom*[tiab]) AND ((VANH[tiab] OR VAMIS[tiab] OR TVNH[tiab] OR (“glove”[tiab] AND “port”[tiab]) OR gloveport[tiab] OR “single port”[tiab] OR “single incision laparoscopic surgery”[tiab] OR SILS[tiab] OR “laparo-endoscopic single site”[tiab] OR “laparoendoscopic single site”[tiab]) OR (“Natural Orifice Endoscopic Surgery”[Mesh] OR NOTES[tiab] OR vNOTES[tiab] OR (“natural”[tiab] AND “orifice”[tiab] AND “endoscop*”[tiab])))

Embase

(‘hysterectomy’/exp OR ‘hysterectom*’:ti,ab,kw OR ‘uterus amputation’:ti,ab,kw OR ‘uterus extirpation’:ti,ab,kw) AND ((VANH:ti,ab,kw OR VAMIS:ti,ab,kw OR TVNH:ti,ab,kw OR (glove:ti,ab,kw AND port:ti,ab,kw) OR gloveport:ti,ab,kw OR ‘single port’:ti,ab,kw OR ‘single incision laparoscopic surgery’:ti,ab,kw OR SILS:ti,ab,kw OR ‘laparo-endoscopic single site’:ti,ab,kw OR ‘laparoendoscopic single site’:ti,ab,kw) OR (‘natural orifice transluminal endoscopic surgery’/exp OR NOTES:ti,ab,kw OR vNOTES:ti,ab,kw OR (natural:ti,ab,kw AND orifice:ti,ab,kw AND endoscop*:ti,ab,kw)))

Cochrane Central

([mh “Hysterectomy”] OR (hysterectom*):ti,ab,kw) AND ([mh “Natural Orifice Endoscopic Surgery”] OR (VANH OR VAMIS OR TVNH OR (“glove” AND “port”) OR gloveport OR “single port” OR “single incision laparoscopic surgery” OR SILS OR “laparo-endoscopic single site” OR “laparoendoscopic single site” OR (Natural AND Orifice AND Endoscop*) OR NOTES OR vNOTES):ti,ab,kw)

Web of Science (All Databases)

(TS = (hysterectom*)) AND (TS = ((Natural AND Orifice AND Endoscop*) OR “NOTES” OR “vNOTES”))

Clinicaltrials.gov

‘Other terms’: hysterectomy (filter: studies with results)

ICTRP WHO

Hysterectomy (filter: with results)

LILACS

tw:((tw:(hysterectomy)) AND (tw:((tw:(natural)) AND (tw:(orifice))))) AND (db:(“IBECS” OR “LILACS” OR “CUMED”))

INAHTA

“hysterectomy”[mh] OR hysterectom* (filter: completed)

Open Grey

Hysterectom*

## References

[1] Santos B.F., Hungness E.S. (2011). Natural orifice translumenal endoscopic surgery: Progress in humans since white paper. World J. Gastroenterol..

[2] Baekelandt J., De Mulder P.A., Roy I.L., Mathieu C., Laenen A., Enzlin P., Weyers S., Mol B.W.J., Bosteels J.J.A. (2017). Postoperative outcomes and quality of life following hysterectomy by natural orifice transluminal endoscopic surgery (NOTES) compared to laparoscopy in women with a non-prolapsed uterus and benign gynaecological disease: A systematic review and meta-analysis. Eur. J. Obstet. Gynecol. Reprod. Biol..

[3] Su H., Yen C.-F., Wu K.-Y., Han C.-M., Lee C.-L. (2012). Transvaginal natural orifice transluminal endoscopic surgery (NOTES) for hysterectomy: Feasibility report of an innovative approach. J. Minim. Invasive Gynecol..

[4] Aarts J.W.M., Nieboer T.E., Johnson N., Tavender E., Garry R., Mol B.W.J., Kluivers K.B. (2015). Surgical approach to hysterectomy for benign gynaecological disease. Cochrane Database Syst. Rev..

[5] Moen M.D., Noone M.B., Elser D.M. (2008). Natural orifice hysterectomy. Int. Urogynecology J. Pelvic Floor Dysfunct..

[6] Baekelandt J. (2015). Notes hysterectomy: A new approach to hysterectomy via natural orifice transluminal endoscopic surgery. Gynecol. Surg..

[7] Higgins J.P.T., Thomas J., Chandler J., Cumpston M., Li T., Page M.J., Welch V.A. (2020). Cochrane Handbook for Systematic Reviews of Interventions Version 6.1 (Updated September 2020).

[8] Moher D., Liberati A., Tetzlaff J., Altman D.G., the PRISMA Group (2009). Preferred reporting items for systematic reviews and meta-analyses: The PRISMA statement. J. Clin. Epidemiol..

[9] Clavien P.A., Barkun J., de Oliveira M.L., Vauthey J.N., Dindo D., Schulick R.D., de Santibañes E., Pekolj J., Slankamenac K., Bassi C. (2009). The Clavien-Dindo classification of surgical complications: Five-year experience. Ann. Surg..

[10] Dindo D., Demartines N., Clavien P.A. (2004). Classification of surgical complications: A new proposal with evaluation in a cohort of 6336 patients and results of a survey. Ann. Surg..

[11] Sterne J.A.C., Savović J., Page M.J., Elbers R.G., Blencowe N.S., Boutron I., Cates C.J., Cheng H.-Y., Corbett M.S., Eldridge S.M. (2019). RoB 2: A revised tool for assessing risk of bias in randomised trials. BMJ.

[12] Sterne J.A., Hernán M.A., Reeves B.C., Savović J., Berkman N.D., Viswanathan M., Henry D., Altman D.G., Ansari M.T., Boutron I. (2016). ROBINS-I: A tool for assessing risk of bias in non-randomised studies of interventions. BMJ.

[13] Baekelandt J.F., De Mulder P.A., Le Roy I., Mathieu C., Laenen A., Enzlin P., Weyers S., Mol B., Bosteels J. (2019). Hysterectomy by transvaginal natural orifice transluminal endoscopic surgery versus laparoscopy as a day-care procedure: A randomised controlled trial. BJOG.

[14] ChiCtr (2019). Hysterectomy by Transumbilical Laparoendoscopic Single Site Surgery(TU-LESS) or Transvaginal Natural Orifice Transluminal Endoscopic Surgery(vNOTES): A Randomised Controlled Trial. http://www.who.int/trialsearch/Trial2.aspx?TrialID=ChiCTR1900023242.

[15] Kct (2020). Vaginal Natural Orifice Transluminal Endoscopic Surgery vs. Single Port Assess for Hysterectomy. http://www.who.int/trialsearch/Trial2.aspx?TrialID=KCT0004605.

[16] Wang C.-J., Huang H.-Y., Huang C.-Y., Su H. (2015). Hysterectomy via transvaginal natural orifice transluminal endoscopic surgery for nonprolapsed uteri. Surg. Endosc. Other Interv. Tech..

[17] Yang Y.S., Kim S.Y., Hur M.H., Oh K.Y. (2014). Natural orifice transluminal endoscopic surgery-assisted versus single-port laparoscopic-assisted vaginal hysterectomy: A case-matched study. J. Minim. Invasive Gynecol..

[18] Kim S.H., Jin C.H., Hwang I.T., Park J.S., Shin J.H., Kim D.W., Seo Y.S., Sohn J.N., Yang Y.S. (2018). Postoperative outcomes of natural orifice transluminal endoscopic surgery-assisted vaginal hysterectomy and conventional laparoscopic-assisted vaginal hysterectomy: A comparative study. Obstet. Gynecol. Sci..

[19] Kaya C., Alay I., Cengiz H., Yıldız G.O., Baghaki H.S., Yasar L. (2020). Comparison of hysterectomy cases performed via conventional laparoscopy or vaginally assisted natural orifice transluminal endoscopic surgery: A paired sample cross-sectional study. J. Obstet. Gynaecol..

[20] Yang C.-Y., Shen T.-C., Lin C.-L., Chang C.Y.-Y., Huang C.-C., Lin W.-C. (2020). Surgical outcomes of hysterectomy by transvaginal natural orifice transluminal endoscopic surgery (vNOTES) compared with laparoscopic total hysterectomy (LTH) in women with non-prolapsed and benign uterine diseases. Taiwan. J. Obstet. Gynecol..

