# Supplementary figures and images for: Systematic Review and Meta-Analysis on Hysterectomy by Vaginal Natural Orifice Transluminal Endoscopic Surgery (vNOTES) Compared to Laparoscopic Hysterectomy for Benign Indications
Source: J Clin Med. 2020 Dec 7;9(12):3959. doi: 10.3390/jcm9123959 (PMC7762322; doi:10.3390/jcm9123959)

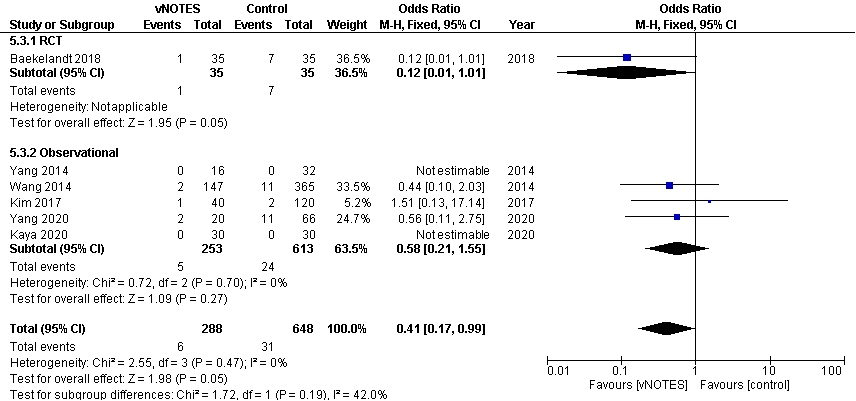

Supplement: Supplementary file 1 [file jcm-09-03959-s001.zip › Figure S1.jpg]

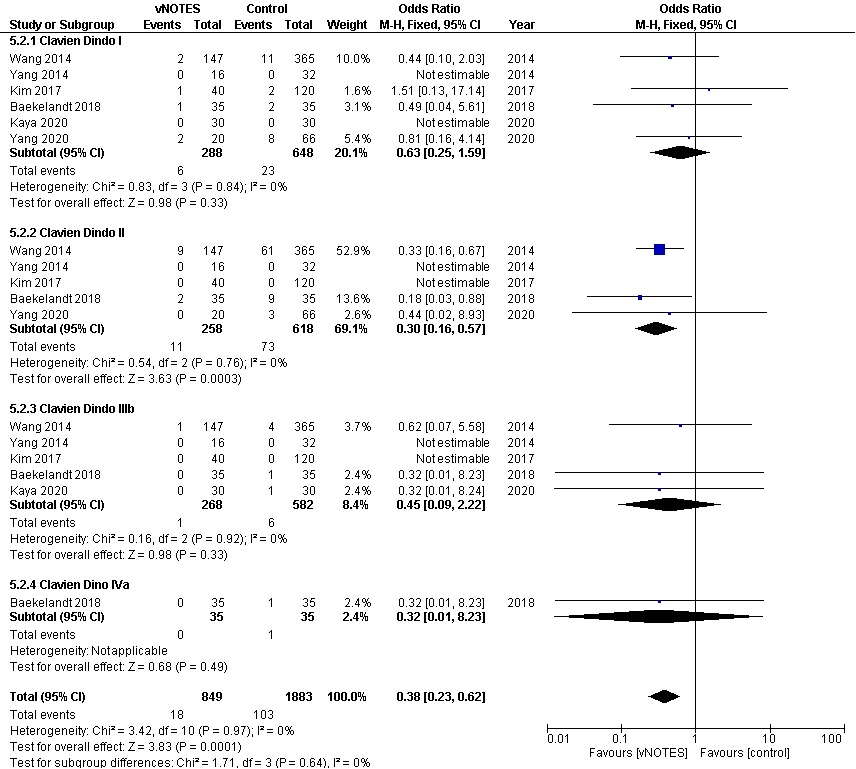

Supplement: Supplementary file 1 [file jcm-09-03959-s001.zip › Figure S2.jpg]

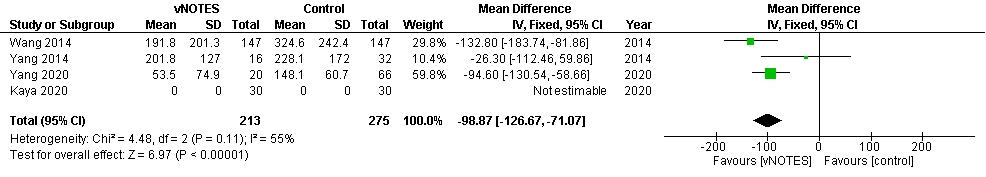

Supplement: Supplementary file 1 [file jcm-09-03959-s001.zip › Figure S3.jpg]

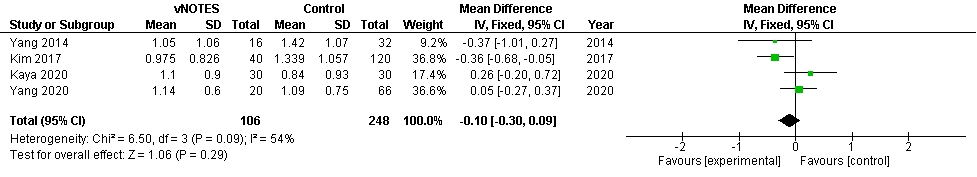

Supplement: Supplementary file 1 [file jcm-09-03959-s001.zip › Figure S4.jpg]

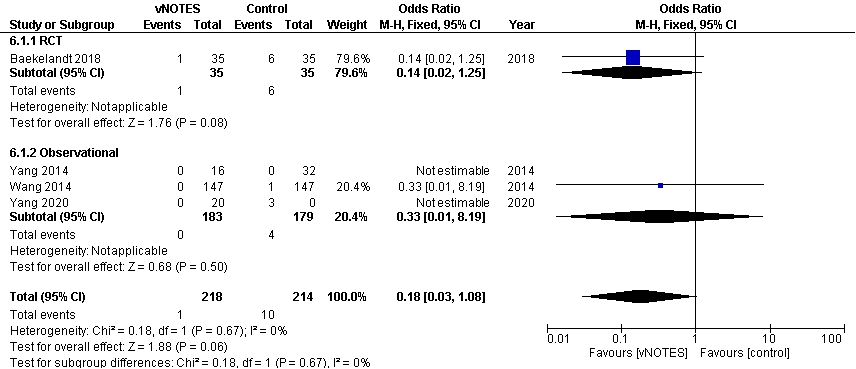

Supplement: Supplementary file 1 [file jcm-09-03959-s001.zip › Figure S5.jpg]
